# Supplementary material for: Genomic analysis of a pre-elimination Malaysian Plasmodium vivax population reveals selective pressures and changing transmission dynamics
Source: Nat Commun. 2018 Jul 3;9:2585. doi: 10.1038/s41467-018-04965-4 (PMC6030216; doi:10.1038/s41467-018-04965-4)
Supplement: Supplementary file 1 — Supplementary Information [file 41467_2018_4965_MOESM1_ESM.pdf]

Supplementary Information

Supplementary Figures

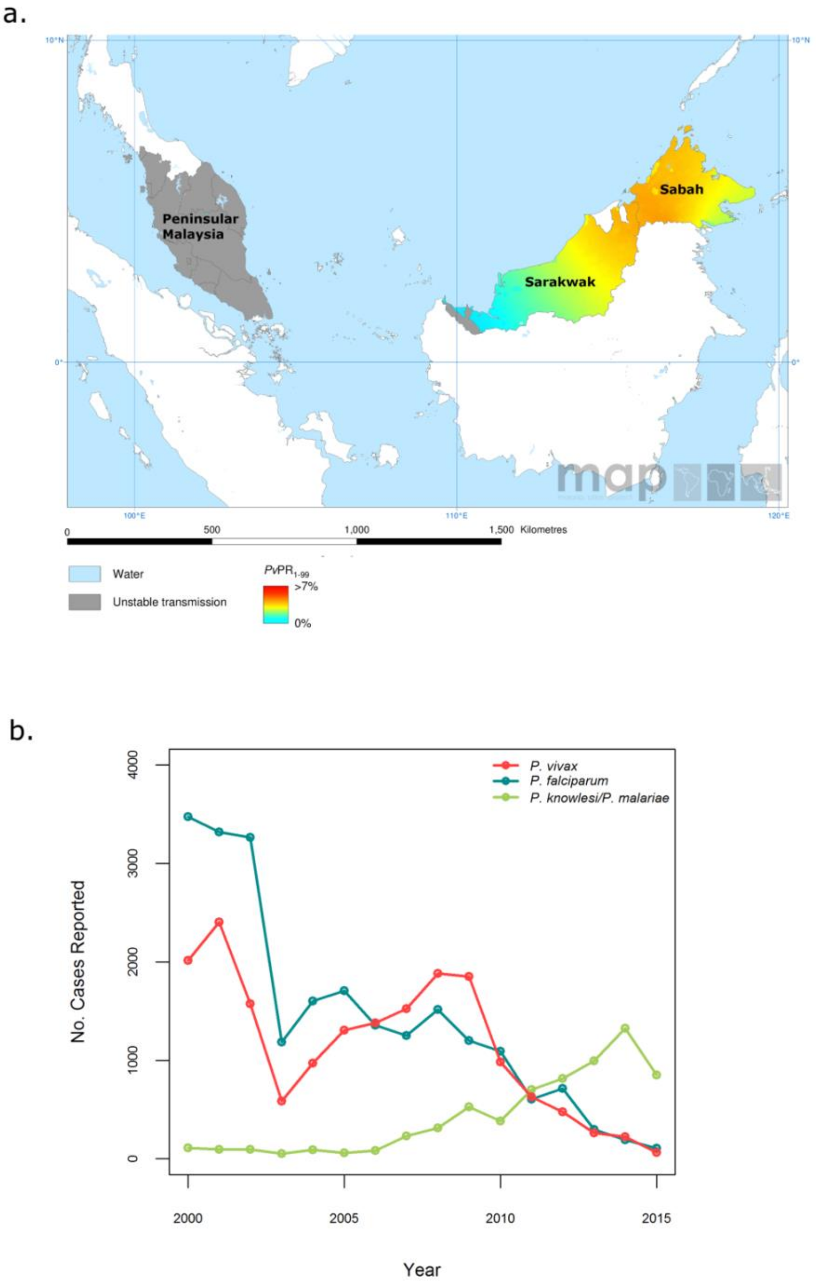

Supplementary Figure 1. *P. vivax* prevalence and incidence in Sabah. Panel a) provides a *P. vivax* prevalence map for Malaysia generated by the Malaria Atlas Project. The colour scales reflect the model-based geostatistical point estimates of the annual mean *P. vivax* parasite rate in the 1-99 year

age range ($Pv\mathrm{PR}_{1\text{-}99}$) within the stable spatial limits of transmission in 2010. All Malaria Atlas Project maps are available to users under the Creative Commons Attribution License CCAL 3.0. [http://www.map.ox.ac.uk/about-map/open-access/]. Panel b) illustrates the annual number of reported cases of *P. vivax*, *P. falciparum* and *P. knowlesi*/*P. malariae* in Sabah between 2000 and 2015. The data was provided by the Sabah Department of Health. Based on population size data provided by the Malaysian Department of Statistics, the annual *P. vivax* incidence (per 1000 population) was approximately 0.78 in 2000, 0.32 in 2010 and 0.02 in 2015.

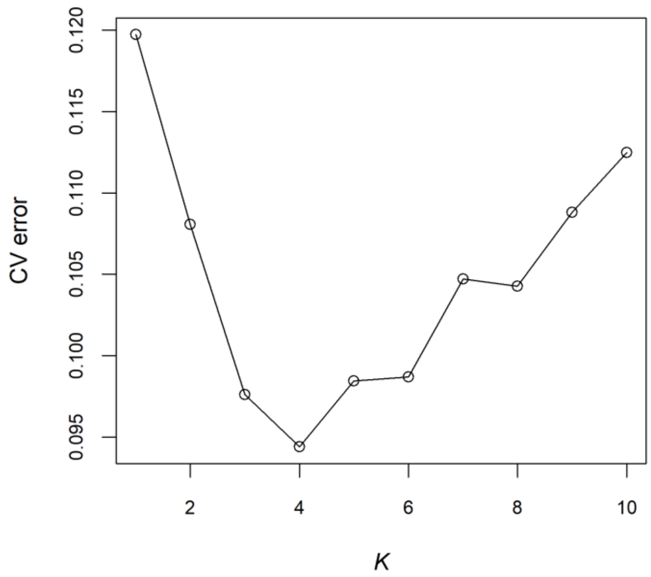

Supplementary Figure 2. Admixture cross-validation errors for estimates of *K* 1 to 10 in the country-wide sample set. Analysis was performed on the full data set (*n*=259 samples). The lowest cross-validation error was observed at *K* = 4 (CV=0.094).

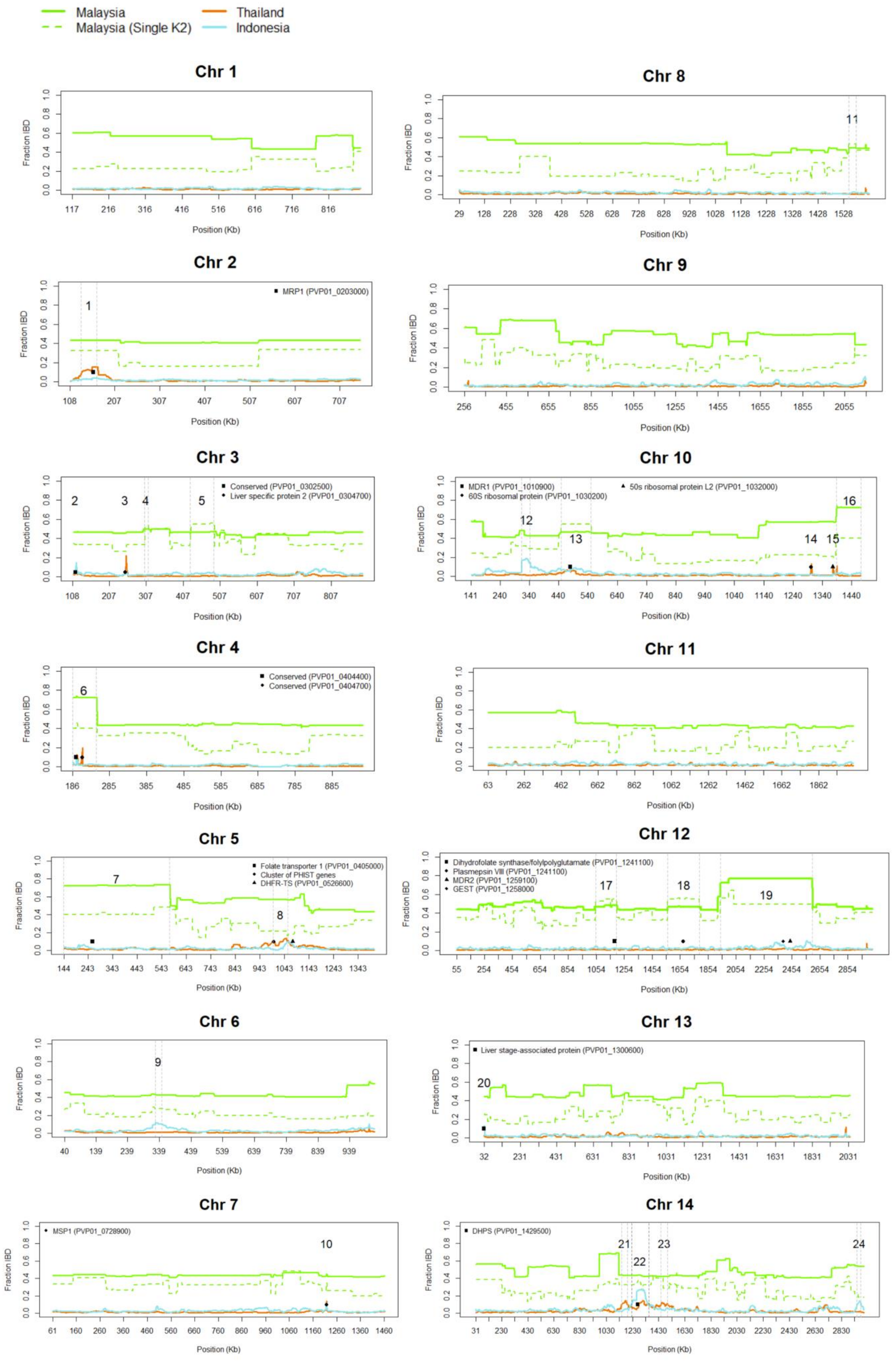

Malaysia
Malaysia (Single K2)
Thailand
Indonesia
Chr 1
Chr 2
MRP1 (PVP01_0203000)
Chr 3
Conserved (PVP01_0302500)
Liver specific protein 2 (PVP01_0304700)
Chr 4
Conserved (PVP01_0404400)
Conserved (PVP01_0404700)
Chr 5
Folate transporter 1 (PVP01_0405000)
Cluster of PHIST genes
DHFR-TS (PVP01_0526600)
Chr 6
Chr 7
MSP1 (PVP01_0728900)
Chr 8
Chr 9
Chr 10
MDR1 (PVP01_1010900)
60S ribosomal protein (PVP01_1030200)
50s ribosomal protein L2 (PVP01_1032000)
Chr 11
Chr 12
Dihydrofolate synthase/folylpolyglutamate (PVP01_1241100)
Plasmepsin VIII (PVP01_1241100)
MDR2 (PVP01_1259100)
GEST (PVP01_1258000)
Chr 13
Liver stage-associated protein (PVP01_1300600)
Chr 14
DHPS (PVP01_1429500)
Fraction IBD
Position (Kb)

Supplementary Figure 3. Fraction of sample pairs that are IBD along each chromosome in Malaysia, Thailand and Indonesia. Each plot presents the fraction of pairwise samples that are IBD for each population at 1 Kb intervals along the accessible (mappable) regions of the given chromosome. Analyses were conducted on monoclonal samples (F_{WS}≥0.95). In Malaysia, analyses were conducted on all samples excluding the 3 putatively imported cases (All) and additionally with single representation of the K2 strain (Single K2). Markedly higher levels of IBD were observed in the Malaysian samples than the Indonesian and Thai samples across the genome. Regions supported by a minimum of two adjacent positions among the top 5% IBD fractions in Malaysia and the top 1% fractions in each of Thailand and Indonesia are numbered from 1-24, with the boundaries of regions exceeding 10 Kb demarked by dashed grey lines. Gene annotations are provided where regions encompass a single gene or a biologically plausible drug resistance candidate. Region 1 (chr2, 126-161 Kb) spans 6 genes; region 2 (chr3, 116-117 Kb) spans 1 gene; region 3 (chr3, 251-252 Kb) spans 1 gene; region 4 (chr3, 303-313 Kb) spans 4 genes; region 5 (chr3, 427-491 Kb) spans 22 genes; region 6 (chr4, 186-250 Kb) spans 13 genes; region 7 (chr5, 144-576 Kb) spans 104 genes; region 8 (chr5, 997-1058 Kb) spans 16 genes; region 9 (chr6, 327-347 Kb) spans 3 genes; region 10 (chr7, 1216-1218 Kb) spans 1 gene; region 11 (chr8, 1550-1577 Kb) spans 6 genes; region 12 (chr10, 314-343 Kb) spans 4 genes; region 13 (chr10, 449-551 Kb) spans 20 genes; region 14 (chr10, 1302-1303 Kb) spans 1 gene; region 15 (chr10, 1376-1377 Kb) spans 1 gene; region 16 (chr10, 1389-1472 Kb) spans 19 genes; region 17 (chr12, 1051-1199 Kb) spans 37 genes; region 18 (chr12, 1566-1792 Kb) spans 48 genes; region 19 (chr12, 1942-2599 Kb) spans 153 genes; region 20 (chr13, 32-33 Kb) spans 1 gene; region 21 (chr14, 1150-1191 Kb) spans 9 genes; region 22 (chr14, 1224-1356 Kb) spans 24 genes; region 23 (chr14, 1449-1499 Kb) spans 19 genes; and region 24 (chr14, 2949-2978 Kb) spans 2 genes.

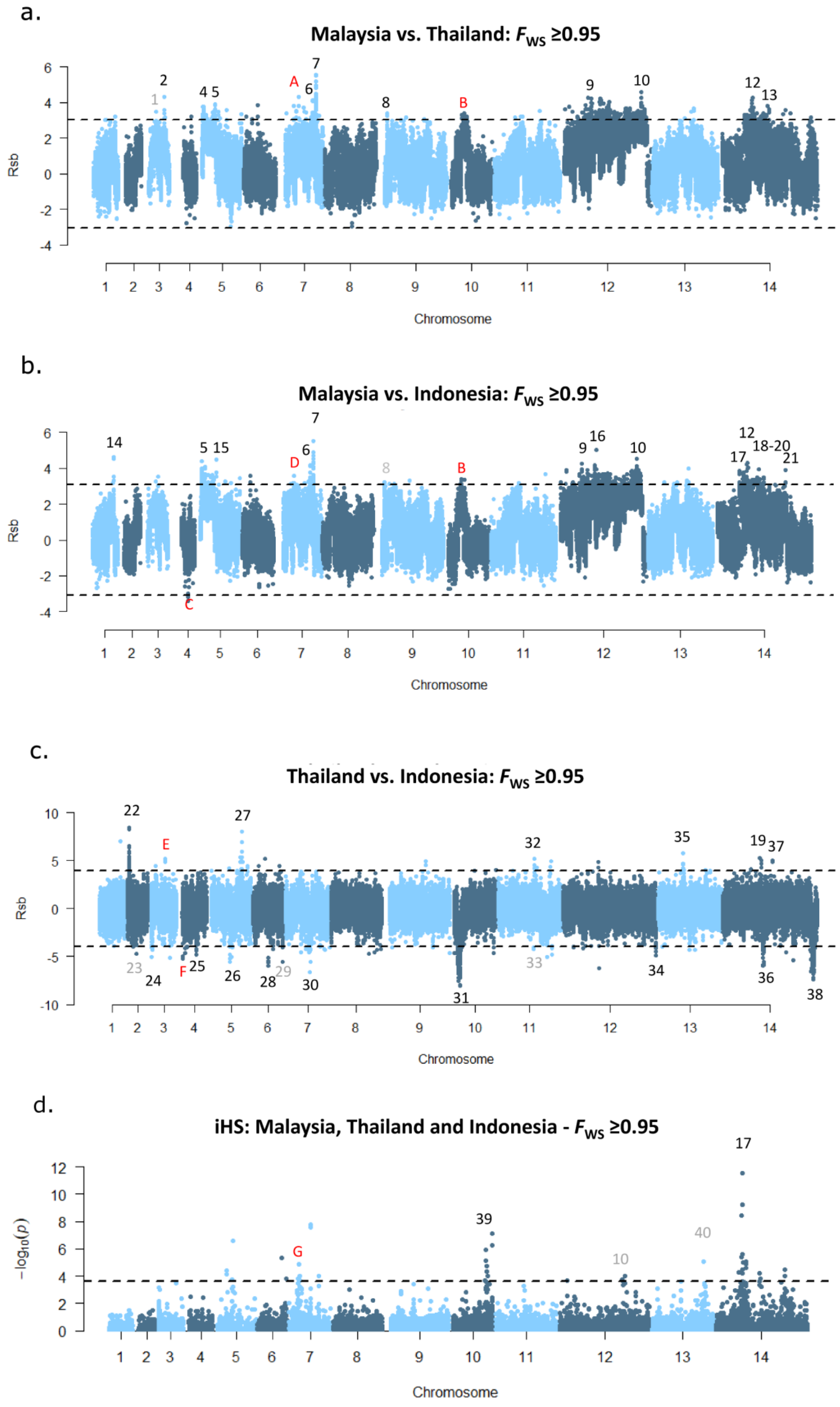

Supplementary Figure 4. Genome-wide scans to identify regions with extended haplotype homozygosity using monoclonal samples. Panels a), b) and c) present Manhattan plots of the *Rsb*

index for the given populations, and panel d) presents the *iHS p-value* for the pooled populations. Analyses were conducted on monoclonal samples (F_{WS}≥0.95). The dashed black lines demark the top 0.5% SNPs with the most significant *p-values*. The dashed grey line in panel d) demarks the top 1% SNPs. Regions with ≥3 SNPs and with 1-2 SNPs within 80 Kb of one another and overall SNP density < 10 Kb per SNP amongst the top 0.5% SNPs that were also identified in the low complexity samples (F_{WS}≥0.60) are numbered in black and grey respectively. Details on these regions are provided in Figure 6. Seven new multi-SNP signals observed in the monoclonal dataset are labelled with red letters A-G. Signals A, B, E, F and G are reflected by 1-2 SNPs in the low-complexity data and hence are represented in Supplementary Tables 6 and 7. Signal C encompasses a conserved gene (PVP01_0415400), and signal D is an intergenic region with no clear candidates. Overall, there are no major differences in the monoclonal and low-complexity datasets.

Supplementary Tables

Supplementary Table 1. Demographic summary of the patients with successfully genotyped samples

Year	No. patients	Median age (Range)	% Males	Median parasite density, μL^{-1} (Range)
2010	24	23 (13-63)	83%	5,642 (929-57,000) [8]
2011	32	20.5 (4-79)	72%	3,152 (120-10,160) [10]
2012	18	30.5 (4-71)	72%	2,124 (179-5,700) [7]
2013	26	11 (2-42)	58%	5,279 (249-24,400)
2014	105	17 (2-68)	69%	3,882 (91-140,500)
2015	7	32 (2-70)	71%	6,767 (77-36250)
**2010-2015**	**212**	**19 (2-79)**	**70%**	**3,995 (77-140,500)** [25]

Superscript denotes number of patients with missing data

Supplementary Table 2. Temporal trends in within-host and population level diversity using the STR genotyping data

Year	No. samples	No. unique MLGs	No. low complexity samples (%)*	No. polyclonal samples (%)	Mean MOI, median (range)	Median *R*s (range)	Median *R*s of unique MLGs (range)	Median *R*s of low complexity samples (range)
2010	24	17	22 (92%)	12 (50%)	1.54, 1.5 (1-3)	4.98 (3.94-8.67)	4.89 (3.90-7.49)	4.93 (3.75-8.73)
2011	32	23	26 (81%)	9 (28%)	1.34, 1 (1-3)	6.51 (4.40-8.65)	6.02 (4.17-7.63)	6.11 (4.45-7.93)
2012	18	16	17 (94%)	3 (17%)	1.17, 1 (1-2)	8.00 (3.89-10.20)	6.67 (3.59-7.59)	7.88 (3.88-9.38)
2013	26	12	25 (96%)	4 (15%)	1.15, 1 (1-2)	3.91 (3.00-5.91)	4.64 (3.00-6.15)	3.60 (2.99-5.94)
2014	105	16	104 (99%)	8 (8%)	1.08, 1 (1-2)	2.51 (2.21-4.22)	4.46 (3.48-6.21)	2.37 (2.16-4.14)

*No. samples with a maximum of 1 multi-allelic locus

Supplementary Table 3. Temporal trends in linkage disequilibrium using the STR genotyping data

Year	No. samples with complete MLGs	$\bar{I}^{S}_{A}$ on all complete MLGs	No. unique MLGs [1]	$\bar{I}^{S}_{A}$ unique MLGs	No. low complexity samples [2]	$\bar{I}^{S}_{A}$ low complexity MLGs
2010	16	0.486 **	11	0.230 **	15	0.529 **
2011	27	0.478 **	18	0.116 **	21	0.613 **
2012	13	0.167 **	11	0.019 *	12	0.179 **
2013	21	0.536 **	9	0.258 **	21	0.536 **
2014	103	0.366 **	15	0.285 **	102	0.373 **

[1] No. unique MLGs in the samples with complete genotyping data. [2] No. samples with a maximum of one multi-allelic locus with complete MLGs. (Monte Carlo simulation, *$p < 0.05$. **$p < 0.01$).

Supplementary Table 4. Demographic details of Individuals with 'outbreak' *P. vivax* strains relative to other cases

	K2 (MLG38)	[s] Other high frequency MLGs	[b] Low frequency MLGs
**Cases, *n***	68	52	67
**Age in years, median (range)**	15 (2-68)	18 (3-45)	24 (2-79)
**Males, *n* (%)**	42 (62%)	38 (73%)	49 (73%)
**Parasite density (μl^{-1}), median (range)**	4,180 (77-140,500)	3,690 (91-42,520) [5]	4,186 (120-57,000) [15]
**Unknown occupation (missing data), *n* (%)**	0/68 (0%)	1/52 (2%)	4/67 (11%)
**Farmer, rubber tapper, forestry or plantation worker**	20/68 (29%)	22/51 (43%)	23/63 (37%)
**Student, *n* (%)**	25/68 (37%)	14/51 (27%)	10/63 (16%)
**Other occupation, *n* (%)**	10/68 (15%)	4/51 (8%)	21/63 (33%)
**Unemployed, *n* (%)**	13/68 (19%)	11/51 (22%)	9/63 (14%)

[a] MLGs observed in 5 or more Independent cases (excluding K2 (MLG38)), including MLGs 39, 43, 44, 45, 46, 49, 50, 52, 55. [b] MLGs observed in less than 5 Independent cases. [5-20] Number of Individuals with missing data.

Supplementary Table 5. European Nucleotide Archive run accession codes for the new *P. vivax* sequence data from Malaysia

Sample ID	Run accession	URL
PY0002-C	ERR054089	[https://www.ebi.ac.uk/ena/data/view/ERR054089]
PY0004-C	ERR152414	[https://www.ebi.ac.uk/ena/data/view/ERR152414]
PY0005-C	ERR152415	[https://www.ebi.ac.uk/ena/data/view/ERR152415]
PY0018-C	ERR527337	[https://www.ebi.ac.uk/ena/data/view/ERR527337]
PY0019-C	ERR527363	[https://www.ebi.ac.uk/ena/data/view/ERR527363]
PY0023-C	ERR1138855	[https://www.ebi.ac.uk/ena/data/view/ERR1138855]
PY0024-C	ERR1138856	[https://www.ebi.ac.uk/ena/data/view/ERR1138856]
PY0026-C	ERR1138857	[https://www.ebi.ac.uk/ena/data/view/ERR1138857]
PY0027-C	ERR1138858	[https://www.ebi.ac.uk/ena/data/view/ERR1138858]
PY0034-C	ERR1138861	[https://www.ebi.ac.uk/ena/data/view/ERR1138861]
PY0035-C	ERR1138862	[https://www.ebi.ac.uk/ena/data/view/ERR1138862]
PY0042-C	ERR1138864	[https://www.ebi.ac.uk/ena/data/view/ERR1138864]
PY0044-C	ERR1138865	[https://www.ebi.ac.uk/ena/data/view/ERR1138865]
PY0045-C	ERR1138866	[https://www.ebi.ac.uk/ena/data/view/ERR1138866]
PY0048-C	ERR1138867	[https://www.ebi.ac.uk/ena/data/view/ERR1138867]
PY0050-C	ERR1138868	[https://www.ebi.ac.uk/ena/data/view/ERR1138868]
PY0051-C	ERR1106842	[https://www.ebi.ac.uk/ena/data/view/ERR1106842]
PY0053-C	ERR1138869	[https://www.ebi.ac.uk/ena/data/view/ERR1138869]
PY0054-C	ERR1138870	[https://www.ebi.ac.uk/ena/data/view/ERR1138870]
PY0055-C	ERR1138871	[https://www.ebi.ac.uk/ena/data/view/ERR1138871]
PY0056-C	ERR1138872	[https://www.ebi.ac.uk/ena/data/view/ERR1138872]
PY0057-C	ERR1138873	[https://www.ebi.ac.uk/ena/data/view/ERR1138873]
PY0058-C	ERR1106843	[https://www.ebi.ac.uk/ena/data/view/ERR1106843]
PY0060-C	ERR1138875	[https://www.ebi.ac.uk/ena/data/view/ERR1138875]
PY0061-C	ERR1138876	[https://www.ebi.ac.uk/ena/data/view/ERR1138876]
PY0067-C	ERR1138879	[https://www.ebi.ac.uk/ena/data/view/ERR1138879]
PY0068-C	ERR1106846	[https://www.ebi.ac.uk/ena/data/view/ERR1106846]
PY0072-C	ERR1138881	[https://www.ebi.ac.uk/ena/data/view/ERR1138881]
PY0073-C	ERR1138882	[https://www.ebi.ac.uk/ena/data/view/ERR1138882]
PY0074-C	ERR1138883	[https://www.ebi.ac.uk/ena/data/view/ERR1138883]
PY0075-C	ERR1138884	[https://www.ebi.ac.uk/ena/data/view/ERR1138884]
PY0076-C	ERR1138885	[https://www.ebi.ac.uk/ena/data/view/ERR1138885]
PY0085-C	ERR1475425	[https://www.ebi.ac.uk/ena/data/view/ERR1475425]
PY0087-C	ERR1475427	[https://www.ebi.ac.uk/ena/data/view/ERR1475427]
PY0088-C	ERR1475395	[https://www.ebi.ac.uk/ena/data/view/ERR1475395]
PY0089-C	ERR1475451	[https://www.ebi.ac.uk/ena/data/view/ERR1475451]
PY0090-C	ERR1475441	[https://www.ebi.ac.uk/ena/data/view/ERR1475441]
PY0092-C	ERR1475396	[https://www.ebi.ac.uk/ena/data/view/ERR1475396]
PY0093-C	ERR1475397	[https://www.ebi.ac.uk/ena/data/view/ERR1475397]
PY0094-C	ERR1475398	[https://www.ebi.ac.uk/ena/data/view/ERR1475398]
PY0096-C	ERR1475399	[https://www.ebi.ac.uk/ena/data/view/ERR1475399]

PY0099-C	ERR1475418	[https://www.ebi.ac.uk/ena/data/view/ERR1475418]
PY0100-C	ERR1475419	[https://www.ebi.ac.uk/ena/data/view/ERR1475419]
PY0101-C	ERR1475420	[https://www.ebi.ac.uk/ena/data/view/ERR1475420]
PY0103-C	ERR1475429	[https://www.ebi.ac.uk/ena/data/view/ERR1475429]
PY0105-C	ERR1475430	[https://www.ebi.ac.uk/ena/data/view/ERR1475430]
PY0107-C	ERR1475439	[https://www.ebi.ac.uk/ena/data/view/ERR1475439]
PY0114-C	ERR1475456	[https://www.ebi.ac.uk/ena/data/view/ERR1475456]
PY0117-C	ERR1475437	[https://www.ebi.ac.uk/ena/data/view/ERR1475437]
PY0119-C	ERR1475457	[https://www.ebi.ac.uk/ena/data/view/ERR1475457]
PY0120-C	ERR1475434	[https://www.ebi.ac.uk/ena/data/view/ERR1475434]
