# Supplementary material for: Genomic analysis of a pre-elimination Malaysian Plasmodium vivax population reveals selective pressures and changing transmission dynamics
Source: Nat Commun. 2018 Jul 3;9:2585. doi: 10.1038/s41467-018-04965-4 (PMC6030216; doi:10.1038/s41467-018-04965-4)
Supplement: Supplementary file 3 — Description of Additional Supplementary Files [file 41467_2018_4965_MOESM3_ESM.pdf]

Description of Additional Supplementary Files

Supplementary Data 1. Summary of samples with high-quality genomic data

Supplementary Data 2. Prevalence of non-synonymous variants in orthologues of *P. falciparum* drug resistance candidates. VQSLOD = VQSLOD score. Non-synonymous variants were identified for all positions with a VQSLOD score >0 i.e. including variants that did not pass the threshold (VQSLOD score >3) applied for inclusion in all other population genomic analyses conducted on the data. Amino acid = amino acid change. Ref = reference. Alt = alternative. Alt 1 = alternative 1 (for tri-allelic positions). *Single base deletions. ** Positions with >20% genotyping failures or heterozygote calls.

Supplementary Data 3. Pairwise IBD fractions for each population at each of the 1 Kb intervals along chromosomes 1-14. Regions supported by a minimum of two adjacent positions among the top 5% IBD fractions in Malaysia and the top 1% fractions in each of Thailand and Indonesia are numbered from 1-24 in the last column (IBD in top 5 % MY or top 1% TH or ID). MY = Malaysia (Sabah); TH = Thailand; ID = Indonesia.

Supplementary Data 4. High-level summary of multi-SNP regions with evidence of F_{ST}, *Rsb* or *iHS* signals of selection. [a] MY = Malaysia (Sabah); TH = Thailand; ID = Indonesia. [b] Genomic regions supported by 3 or more of the top 0.5% of SNPs with the lowest *p*-values (*Rsb* and *iHS*) or F_{ST} scores ≥0.8 within 80 Kb of one another and with an overall SNP density less than 10 Kb per SNP. [c] Population with evidence of relatively extended haplotype homozygosity; MY = Malaysia (Sabah); TH = Thailand; ID = Indonesia. [c] Previous studies have that described haplotype-based signals of selection within the given genomic region. *Identified a shorter signal at *MSP7*. **Identified a shorter signal at *MDR2*.

Supplementary Data 5. Summary of positions with F_{ST}≥0.8. F_{ST} scores greater than or equal to 0.8 are highlighted in green, and scores between 0.6 and 0.8 in yellow. MY = Malaysia, TH = Thailand, ID = Indonesia. MY Multi-SNP Signal: regions with a minimum of 3 SNPs with F_{ST}≥0.8 within 80 Kb of one another in comparisons between Sabah and Thailand or Indonesia. VQSLOD = VQSLOD score. Amino acid = amino acid change; "." refers to intergenic sites. RAF = reference allele frequency.

Supplementary Data 6. Summary of the top 0.5% of SNPs with the lowest *Rsb* p-values. MY = Malaysia, TH =Thailand, ID = Indonesia. Top 0.5%: population comparisons within which the SNP is among the top 0.5% with the lowest *p*-values. Signals: regions with a minimum of 3 SNPs within 80 Kb of one another and overall SNP density less than 10 Kb per SNP amongst the top 0.5% SNPs with the lowest *p*-values are labelled. The maximal *Rsb* scores for a given signal are highlighted in green.

Supplementary Data 7. Summary of the top 0.5% and 1% of SNPs with the lowest *iHS* p-values. MY = Malaysia, TH =Thailand, ID = Indonesia. Signals: regions with a minimum of 3 SNPs within 80 Kb of one another and overall SNP density less than 10 Kb per SNP amongst the top 0.5% and 1% of SNPs with the lowest *p*-values are labelled respectively. Signal labels include labels relative to the *Rsb* analysis in parentheses.

Supplementary Data 8. Summary of copy number variants
